# Are we immune by chance?

**DOI:** 10.7554/eLife.32783

**Published:** 2017-11-27

**Authors:** Andrea L Graham, Ann T Tate

**Affiliations:** 1 Department of Ecology and Evolutionary BiologyPrinceton UniversityPrincetonUnited States; 2 Department of Biological SciencesVanderbilt UniversityNashvilleUnited States

**Keywords:** innate immunity, inter-individual variation, stochasticity, mixture modelling, Imd pathway, *D. melanogaster*

## Abstract

The sooner the immune system launches, the greater the chances the host has of survival.

**Related research article** Duneau D, Ferdy JB, Revah J, Kondolf H, Ortiz GA, Lazzaro BP, Buchon N. 2017. Stochastic variation in the initial phase of bacterial infection predicts the probability of survival in *D. melanogaster*. *eLife* **6**:e28298. doi: 10.7554/eLife.28298

Literature and cinema abound with stories of chance encounters that lead to life-changing events. Likewise, in meteorology, the flap of a butterfly’s wing can, in theory, lead to the formation of a tornado thousands of miles away, due to the cascading effects of randomness and stochastic variation. However, the role of chance in immunology has received scant attention.

Our understanding of immune defense has greatly improved over the decades, but one central question still preoccupies scientists: why do people respond to infection in such different ways, when the right response can make the difference between life and death? Several factors are thought to influence immunity, including age, gender or genes. Now, in eLife, David Duneau, Nicolas Buchon and colleagues at Cornell University and the University of Toulouse 3 report that random variations in how long it takes the host’s immune system to react could also play a role in determining if the host can survive an infection (Duneau et al., 2017).

The researchers suggest that whether a host lives or dies is partly a matter of a lucky start. To test this idea, Duneau et al. studied how genetically identical fruit flies, raised in the same environment, responded to a variety of pathogens. The bacteria caused two possible outcomes – death or chronic infection – with significant differences in the fate of individual flies. Moreover, these differences were independent of the amount of pathogen, the age of the host, and the genetic background of both the host and the pathogen.

Duneau et al. developed a mathematical synopsis of their results, which showed that timing was a crucial factor: the sooner the immune system reacted to a potential threat, the higher the chances of survival were. If the immune system responded too late, the bacteria continued to grow until the fly died (Figure 1). Thus, even subtle variations during the early stages of an immune response can lead to stark differences in the chances of dying.

**Figure 1.**
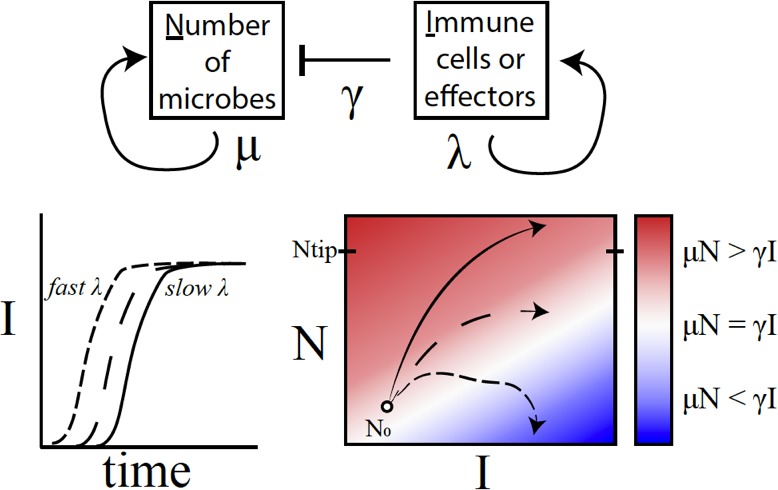
A conceptual framework to explain why variations in immune activation lead to different disease outcomes. In a simple model (top), microbes (N) grow exponentially at rate μ, while each immune cell or effector molecule (I) can kill a microbe at rate γ. The number of immune cells or molecules depends on the rate λ, which in turn depends on a number of factors such as the level of standing immunity, the time taken to activate the immune response (bottom left), and the maximum capacity of the system to produce immune components. The interaction between a given type of microbe and the immune system can be characterised by a map (bottom right) that plots μN – γI as a function of I (horizontal axis) and N (vertical axis). If the growth rate of microbes exceeds the killing capacity of the immune system (μN > γI), the microbes grow exponentially throughout the infection (solid line; red region). If the growth rates become equal (μN = γI), the outcome is a persistent, 'set-point' level of chronic infection (long dashes; white region). If the growth rate of the immune response outpaces that of microbes (μN < γI), the infection will clear up (short dashes; blue) – this was, however, rarely observed in the fruit flies. Duneau et al. suggest that if the number of microbes reaches a certain level (N_tip_), the host will become incapable of producing a sufficient immune response to control the microbes. In this case, the bacteria will continue to grow until the host is dead.

The idea that random variations can have such potent effects may seem surprising and appear to contradict our current knowledge of immunological pathways in both insects and mammals, which are often described as deterministic (e.g., Janeway and Medzhitov, 2002; Lemaitre and Hoffmann, 2007). Qualitatively speaking, they may well be deterministic (in the sense that event A triggers expression of B, which in turn triggers pathogen-killing mechanism C). However, the devil is in the quantitative detail, as we can expect randomness in every step.

For example, the launch of an immune response can sometimes be predicted by mass action, because collisions have to take place between the pathogens and the cells of the immune system (e.g., Sykulev et al., 1995): the stronger the pathogen attack, the higher the immune reaction, on average. Nevertheless, how rapidly immune cells react will vary by host (Frank, 2002), partly because of variation in the rate at which immune cells patrol through the body (e.g., Lee et al., 2012). But pure chance can also influence the likelihood and strength of a response, particularly in hosts exposed to very few pathogens (e.g., Ben-Ami et al., 2010). Subtle variation in the initial density, reactivity and efficacy of immune cells is therefore likely to translate into stochasticity in the induced response.

Duneau et al. provide insight into the role of such chance in host-pathogen interactions. The survival of a fly was linked to the ability of the pathogen to multiply before the fly's defense kicked in. Once the immune system got the upper hand, the pathogen density went down to a certain persistent value that the researchers called the ‘set-point’ (Figure 1). The sooner the immune system succeeded, the greater the chances of survival. However, if the bacterial burden outstripped the immune system, the fly eventually died.

This interplay between random variations and determinism in the early stages of the immune response may well explain other life or death outcomes following changes in diet or repeated exposure to pathogens (e.g., Howick and Lazzaro, 2014; Tate et al., 2017). More generally, the work of Duneau et al. furthers our understanding of the evolutionary processes that drive host-parasite interactions, including natural selection for rapid and accurate inducible defenses (Frank, 2002; Tollrian and Harvell, 1999). We look forward to further work that will help to uncover the mechanisms underlying these fascinating findings. But for now, the role of chance in driving or constraining such processes remains a frontier of science.
